# Buyer and seller data from pay what you want and name your own price laboratory markets

**DOI:** 10.1016/j.dib.2017.04.049

**Published:** 2017-05-04

**Authors:** Florentin Krämer, Klaus M. Schmidt, Martin Spann, Lucas Stich

**Affiliations:** a Department of Economics, University of Munich, D-80539 Munich, Germany; b Munich School of Management, University of Munich, D-80539 Munich, Germany

## Abstract

Pay What You Want (PWYW) and Name Your Own Price (NYOP) are customer-driven pricing mechanisms that give customers (some) pricing power and that have been used in service industries with high fixed costs to price discriminate without setting a reference price. This paper describes buyer and seller data in a series of induced-value laboratory experiments that compare PWYW and NYOP in monopoly and competitive situations. Sellers are in a one-shot interaction with buyers. Sellers using customer-driven pricing mechanisms may exogenously or endogenously receive additional promotional benefits, for instance through word-of-mouth effects. The major findings based on the data presented here are reported in the paper "Delegating Pricing Power to Customers: Pay What You Want or Name Your Own Price?" (Krämer et al., 2017) [3].

**Specifications Table**

**Table:** 

Subject area	*Economics and Marketing*
More specific subject area	*Behavioral Economics, Pricing, Auctions*
Type of data	Subjects’ decisions in induced-value laboratory experiments
How data was acquired	*Sessions conducted at the experimental laboratory of the University of Munich (MELESSA)*
Data format	*.csv files (data and data description)*
Experimental factors	*Customer-driven pricing mechanism (NYOP or PWYW), market structure (monopoly or competition with posted-price seller), seller choice of customer-driven pricing mechanism (flexible or fix), additional benefits (exogenous or endogenous)*
Experimental features	*Subjects can either have the role of buyer or seller and remain in this role throughout the experiment. All treatments are repeated for 20 periods and subjects are randomly rematched every period. Buyers’ valuations, sellers’ costs and values of exogenous benefit (if applicable) are randomly drawn.*
Data source location	*Munich, Germany*
Data accessibility	*Data included in this paper*

**Value of the data**

• This dataset compares buyer and seller behavior in two customer-driven pricing mechanisms: Name Your Own Price and Pay What You Want.
• Researchers can combine the analysis of buyer and seller behavior in one-shot interactions of PWYW and NYOP (this dataset) in comparison to the data related to the paper by Schmidt et al. [4] where buyers and sellers interact repeatedly.
• This dataset provides a baseline on buyer and seller behavior in controlled interactions to compare with in case of related field experiments.

## 1 Data

The data comprise buyer and seller behavior of a total of 384+144 subjects who participated in 8 different treatments of an experiment reported in Krämer et al., 2017: 6 treatments with exogenous benefits (NYOP monopoly, NYOP competition with fixed roles, NYOP competition with a flexible seller; PWYW monopoly, PWYW competition with fixed roles, PWYW competition with a flexible seller) and 2 treatments with endogenous benefits (NYOP, PWYW). The data captures for each treatment, round and subject the experimental conditions, subsequent decisions and outcome.

In the first set of experiments, sessions lasted about two hours and subjects earned on average 18 Euros (about 24 US Dollars at the time of the experiment), including a show-up fee of 4 Euros. In the endogenous benefit treatments average earnings amounted to 25 Euros (about 27 US Dollars at the time of the experiment), including a show-up fee of 4 Euros. All sessions were conducted at the experimental laboratory of the University of Munich (MELESSA). The subject pool consisted mainly of students from a wide range of majors. Treatments were implemented using zTree [1] and subjects were recruited using ORSEE [2].

Table 1 summarizes the data provided with this paper. A brief description of the variables is contained in the Excel file *variable_description_pwyw_nyop.xlsx.*

**Table 1:** Overview of Data.

**Pricing Mechanism**	**Treatment**	**Name of data file**	**Number of variables**
Pay What You Want	Competition Flexible	PCFlex	39
	Competition Fixed	PCFix	33
	Monopoly	PM	26
	Endogenous Benefit	PCEB	39
Name Your Own Price	Competition Flexible	NCFlex	41
	Competition Fixed	NCFix	36
	Monopoly	NM	28
	Endogenous Benefit	NCEB	43

## 2 Experimental design, materials and methods

### 2.1 Basic setup

In each treatment, subjects are randomly assigned to a role (i.e., buyer or seller) that remains fixed throughout the experiment. Each session consists of 24 participants resulting in three markets in the competition treatments (two sellers facing six buyers in each market) and six markets in the monopoly treatments (one seller facing three buyers). All treatments are repeated for 20 periods, and subjects are randomly re-matched every period. The data comprise eight sessions of the competition treatments and another eight sessions of the monopoly treatments. In order to perfectly control the valuations of the buyers and the costs of the sellers we use an induced-value design [5].

Conditional on using a customer-driven pricing mechanism, sellers may receive a benefit (b) per unit sold. In the first set of experiments the benefit is exogenously given. In the second set of experiments, benefits are created endogenously in the lab (see below).

The exogenous benefit is proportional to the number of units sold. In order to identify the effect of b, we assigned a strictly positive benefit b randomly to 50% of all markets and the remaining 50% of markets have a benefit of zero. Sellers know whether they enjoy a positive benefit from using a customer-driven pricing mechanism while customers know only that these benefits exist in half of all cases. At the end of each session we elicit information about risk preferences and social preferences of the participants and their demographic characteristics.

### 2.2 Competition treatments

#### 2.2.1 Competition with flexible roles

In treatments PCFlex (PWYW, Competition, Flexible role) and NCFlex (NYOP, Competition, Flexible role) one of the two sellers can choose whether to use posted prices or to use PWYW (NYOP, respectively) while the other (traditional) seller has to use a posted price. At the beginning of each period all subjects observe the per-unit cost of the good which is the same for both sellers and drawn from c∈{10, 30, 50}. The flexible seller privately learns the per-unit benefit b∈{0, 40} from using a customer-driven pricing mechanism and buyers privately learn their valuations of the good drawn independently from v∈{10, 25, 40, 60, 120, 200}. Then each seller decides whether to enter the market. Additionally, the flexible seller decides which pricing mechanism to use. All buyers and sellers subsequently learn about the market structure. Now the posted-price sellers set their prices, and a NYOP seller sets the (secret) threshold price above which s/he is willing to accept all bids. Finally buyers decide whether and if so from which seller to buy. If they opt for a posted-price seller they have to pay the posted price. If they decide to shop with a PWYW seller they get the good with probability 1 and are free to decide how much to pay for it voluntarily (including a price of zero). If they go for a NYOP seller they are asked to submit a bid. If that bid exceeds the secret threshold price set by the seller in advance, they pay their bid and a transaction occurs. In case the bid is below the seller׳s threshold price, they do not receive the good and do not have to pay either. Finally, payoffs are realized.

#### 2.2.2 Competition with fixed roles

The competition treatments with fixed roles (treatments PCFix and NCFix) are set up identically to the treatments with flexible roles except for the fact that one of the sellers is constrained to use either PWYW (in treatment PCFix) or NYOP (in treatment NCFix) if s/he decides to enter the market.

### 2.3 Monopoly treatments

In two monopoly treatments (PM, PWYW Monopoly) and (NM, NYOP Monopoly) there is only one seller who is forced to use PWYW (NYOP, respectively) if s/he enters the market. In these treatments, a market consists of only three buyers. Costs and benefits are parameterized as in the competition treatments, while buyers’ valuations are drawn from a restricted set, v∈{40, 60, 120, 200}. All sellers have the same cost-benefit combination in a given period as in the competition treatments and learn that combination before market entry.

### 2.4 Endogenous benefit

The exogenous benefit treatments varied our previous designs as follows: Each market consists of two sellers and six buyers. The demand is now split into two groups: There are two well-informed buyers, who are fully aware of the market structure, and four follow-up buyers, who have to rely on word of mouth to learn which seller(s) entered the market. The purchasing decisions of the well-informed buyers directly affect the market structure for the follow-up buyers, modeling word-of-mouth advertising in a reduced fashion. For example, if both well-informed buyers purchase at the PWYW seller, only the PWYW seller is visible to the follow-up buyers. However, follow-up buyers may become fully informed about which sellers are in the market if they pay an additional search cost. Furthermore, in the new NYOP treatment buyers can still buy from the posted-price seller if their submitted bid was not successful.

With the beginning of a new period, buyers and sellers are informed about costs (c ∈ {5, 10, 20, 30, 50}). In addition, buyers learn their valuations (v∈{10, 25, 40, 60, 120, 200}). Then, sellers decide on entry. Having observed the sellers’ entry decisions, the two well-informed buyers decide whether and if so with which seller to transact. If at least one of the well-informed buyers has opted for the PWYW/NYOP seller, this seller is available for follow-up buyers at no additional cost. The same applies for the posted-price sellers. If only one seller is available to follow-up buyers, they can invest search costs (csinvest=10) to find out whether the other seller has entered the market. If this is the case, they can also purchase from the discovered seller. If the market for well-informed buyers is split equally, both sellers are available at no additional search cost. After learning about the market structure follow-up buyers make their purchasing decisions. In case of submitting an unsuccessful bid to a NYOP seller, the buyer can turn to the posted-price seller if this seller is available. Thus, the availability of sellers for follow-up buyers in the NYOP treatment depends on the interaction between the well-informed buyers and the sellers.

To illustrate this consider the following situation: A well-informed buyer submits a bid to the NYOP seller which is rejected. The buyer subsequently purchases the good from the posted-price seller, making both sellers available to the follow-up buyers. However, in all cases where both well-informed buyers interact with the same seller only this seller is available to the follow-up buyers.
