# Supplementary material for: Buyer and seller data from pay what you want and name your own price laboratory markets
Source: Data Brief. 2017 May 4;12:513–7. doi: 10.1016/j.dib.2017.04.049 (PMC5425341; doi:10.1016/j.dib.2017.04.049)
Supplement: Supplementary file 1 — Supplementary material [file mmc1.docx]

**Statement concerning Conflicts of Interest**

We wish to confirm that there are no known conflicts of interest associated with this publication and there has been no significant financial support for this work that could have influenced its outcome.
